# Utility of C-Reactive Protein Levels for Early Prediction of Dengue Severity in Adults

**DOI:** 10.1155/2015/936062

**Published:** 2015-07-12

**Authors:** Chien-Chih Chen, Ing-Kit Lee, Jien-Wei Liu, Shi-Yu Huang, Lin Wang

**Affiliations:** ^1^Department of Emergency Medicine, Kaohsiung Chang Gung Memorial Hospital, Kaohsiung 833, Taiwan; ^2^Division of Infectious Diseases, Department of Internal Medicine, Kaohsiung Chang Gung Memorial Hospital, Kaohsiung 833, Taiwan; ^3^Chang Gung University Medical College, Tao-Yuan 333, Taiwan; ^4^Department of Pediatric, Kaohsiung Chang Gung Memorial Hospital, Kaohsiung 833, Taiwan

## Abstract

Dengue has broad clinical presentation with unpredictable clinical evolution and outcome. We aimed to evaluate the utility of C-reactive protein (CRP) levels for distinguishing between mild and severe cases in the early phase of the dengue illness. We retrospectively evaluated adults with dengue from 2006 to 2014, according to 1997 and 2009 World Health Organization (WHO) criteria for severity. Of 191 included patients, 32.9% had nonshock dengue hemorrhagic fever (DHF), 3.1% dengue shock syndrome (DSS), and 7.9% severe dengue. The risk of DHF/DSS and severe dengue is significantly related to the increasing levels of CRP. Of 191 patients, 97 had CRP levels measured during the febrile (days 1–3); 85 during the critical (days 4–6); and 9 during the convalescent (days 7–10) illness phases. During the febrile phase, there was significant higher CRP level for DSS versus DF/nonshock DHF and severe dengue versus nonsevere dengue, with CRP cutoff level 30.1 mg/L (area under the receiver operating characteristic curve (AUC), 0.938; 100% sensitivity, 76.3% specificity) and 24.2 mg/L (AUC, 0.717; 70% sensitivity, 71.3% specificity), respectively. Our study highlights the utility of the CRP levels in early prediction of DSS and severe dengue in adult patients.

## 1. Introduction

Dengue is among the most significant arthropod-borne viral diseases in the world [1]. Clinically, dengue manifestation ranges from nonspecific febrile illness, dengue fever (DF), and dengue hemorrhagic fever (DHF), to the more severe form of dengue shock syndrome (DSS), according to 1997 World Health Organization (WHO) classification [2]. Limitations have been reported regarding its complexity and applicability [3, 4], and the 2009 WHO revised classification divided dengue-affected patients into two categories: nonsevere (with and without warning signs) and severe dengue [5]. The cornerstone of management of patients with dengue and prevention of dengue-related mortality is early recognition of severe cases and timely management [6, 7]. Conversely, clinically differentiating severe and mild forms of the dengue illness is difficult during the early phase of the infection [5, 8, 9]. Several studies have explored this question and tried using clinical and laboratory tests such as white blood cell counts, serum protein, and urea to predict severe dengue [9, 10]. Nevertheless, dengue has broad clinical presentation with unpredictable clinical evolution and outcome. As a result, patients with dengue are often admitted for close monitoring, which can overload the health care system, particularly in resource-limited circumstances. Warning signs (abdominal pain or tenderness, persistent vomiting, clinical fluid accumulation, mucosal bleed, lethargy or restlessness, liver enlargement >2 cm, and an increase in the hematocrit concurrent with a rapid decrease in the platelet count) proposed by the 2009 WHO criteria are considered as potentially key factors for early recognition of severe dengue [5]; however, the sensitivity of each warning sign in predicting severe dengue was reportedly extremely low [8]. A reliable and useful simple biomarker to distinguish between severe and mild illness of dengue during the early phase of infection is helpful to the treating physician in making the decision as to whether or not to admit the patient for management. C-reactive protein (CRP) is an acute-phase protein that is synthesized by the liver within six hours of the onset of inflammation [11]. Illnesses that usually cause obvious changes in CRP levels include infection, burns, trauma, inflammatory disease, and malignancy [11–13]. Clinically, CRP often used to help distinguish viral infections from bacterial infections or to monitor the response to therapy [14]. CRP has also been assessed as a biomarker to discriminate between dengue and malaria [15]. In one study, CRP levels were significantly higher in patients with DHF than in those with DF [16]. This finding showed that the degree of CRP levels might indicate the severity of dengue infection. However, there has not been a study evaluating the use of CRP levels to distinguish between mild and severe dengue during the early phase of infection. The aim of our study was to evaluate the use of CRP levels in predicting severe illness of dengue at the early phase of infection. Our findings may be valuable for clinicians working in crowded emergency rooms, particularly in resource-poor settings.

## 2. Materials and Methods

### 2.1. Ethics Statement

The study was approved by the Institutional Review Board of Chang Gung Memorial Hospital (CGMH) (document number 104-2250B) with a waiver of informed consent for the collection of data.

### 2.2. Settings and Participants

Adult patients (≥18 years of age) with laboratory-confirmed dengue virus (DENV) infection, admitted between 2006 and 2014 at Kaohsiung CGMH, a 2,700-bed primary care and tertiary referral medical center in southern Taiwan, were included in this retrospective study. We excluded patients with dengue concurrent with bacteremia and those clinically diagnosed with superimposed bacterial infections. All patients received medical treatment at the discretion of their attending physicians. Demographic, clinical, and laboratory information of the patients was retrieved from medical records for analyses.

### 2.3. Categories of Dengue Illness Severity

Patients were separately categorized using clinical and laboratory data from the entire clinical course as (i) DF, nonshock DHF, or DSS, according to the 1997 WHO case classification [2], and (ii) nonsevere dengue (with and without warning signs) or severe dengue, based on the 2009 WHO case definitions [5]. Patients were diagnosed as DHF if they had fever, thrombocytopenia, bleeding, and evidence of plasma leakage (i.e., presence of hemoconcentration, pleural effusion, ascites, and/or hypoalbuminemia) [2]. DHF patients with circulatory failure were categorized as DSS [2]. Severe dengue is defined as evidence of plasma leakage associated with shock or respiratory distress, severe bleeding, or severe organ involvement [5].

### 2.4. Laboratory Methods

Acute and convalescent serum samples from dengue patients were obtained. Detection and amplification of DENV ribonucleic acid were performed using DENV-specific real-time reverse transcription polymerase chain reaction (RT-PCR; QuantiTect SYBR Green RT-PCR Kit; QIAGEN GmbH, Hilden, Germany) as previously described [17]. The DENV-specific immunoglobulin G (IgG) antibodies were assessed by the IgG capture enzyme-linked immunosorbent assay (Panbio, Queensland, Australia) [18]. Dengue nonstructural glycoprotein-1 antigen was detected with the Platelia Dengue NS1Ag-ELISA (Bio-Rad Laboratories, Marnes-la-Coquette, France) according to the manufacturer's instructions [19, 20]. The laboratory diagnosis of DENV infection was made based on either a positive DENV-specific RT-PCR, a positive DENV-specific nonstructural glycoprotein-1 antigen in acute-phase serum, or a fourfold increase in DENV-specific IgG antibody in convalescent serum compared with the acute phase as previously described [6, 7].

Patients had CRP levels measured at presentation or during hospitalization at the physician's discretion. Levels of CRP were measured by means of a highly sensitive turbidimetric immunoassay using a monoclonal antibody to CRP coated on polystyrene beads with a lower limit of detection of 0.2 mg/L (SYNCHRON CX systems) [21, 22]. A reference value of CRP < 5.0 mg/L was determined in this study.

### 2.5. Statistical Analysis

CPR data was presented as median and range. We assessed the clinical relevance of elevated CRP level based on the severity of dengue in accordance with the 1997 and 2009 WHO dengue classification [2, 5], respectively. Further, we conducted subgroup analysis for CRP levels stratified by the febrile (days 1–3 of illness), critical (days 4–6 of illness), and convalescent (days 7–10 of illness) phases as defined by 2009 WHO criteria and compared across dengue severity classification according to 1997 and 2009 WHO criteria. Mann-Whitney* U* test was used to determine statistical significance for continuous variables. Finally, to assess the utility of CRP levels to predict the occurrence of DSS and severe dengue, we use receiver operating characteristic curve (ROC) to determine the cutoff level of CRP for DSS and severe dengue, respectively. The area under the curve (AUC) between 0.90–1, 0.80–0.90, 0.70–0.80, 0.60–0.70, and 0.50–0.60 was defined as excellent, good, fair, poor, and failing, respectively. The sensitivity and specificity of the cutoff CRP level were calculated.

## 3. Results

### 3.1. Patient Characteristics

During the study period, 191 adult patients with dengue (mean age, 52.6 years; males, 47.6%) were included. The mean duration of illness prior to hospital presentation was 3.4 (±1.7) days. Using the 1997 WHO classification, 63.9% had DF, 32.9% nonshock DHF, and 3.1% DSS. Using the 2009 WHO criteria, 92.1% had nonsevere dengue and 7.9% severe dengue. Of the overall 90 DENV serotypes identified, DENV-2 accounted for 70%, followed by DENV-2 (24.4%) and DENV-1 (5.6%). Among the 191 patients, the mean interval from dengue onset to blood sampling for CRP was 3.4 (±1.7) days and the mean CRP level was 19.3 (±27.6) mg/L. Of the 191 patients, 3 DSS (severe dengue) were fatal. Demographic, clinical, and laboratory information is summarized in Table 1.

### 3.2. Dengue Severity and CRP Level

For patients with DF, nonshock DHF, and DSS, median CRP levels were 8.5 mg/L, 15.2 mg/L, and 124.5 mg/L, respectively (Table 2). The CRP level was significantly higher for nonshock DHF compared to DF (*P* = 0.024), DSS compared to DF (*P* < 0.001), DSS compared to nonshock DHF (*P* < 0.001), as well as DSS compared to DF/nonshock DHF (*P* = 0.011). By the 2009 WHO dengue severity classification, median CPR levels for nonsevere dengue and severe dengue were 9.8 mg/L and 30.7 mg/L, respectively (Table 3). When compared to patients with nonsevere dengue, patients with severe dengue had significantly higher CRP levels (*P* = 0.009).

Six patients (median age, 64.5 years [range, 18–85]; males, 50%) had extremely high CRP levels (greater than 100 mg/L). Of these 6 patients, 1 (16.7%), 1 (16.7%), and 4 (66.7%) were classified, according to the WHO 1997 criteria, as having DF, grade 1 DHF, and DSS, respectively, whereas 5 (83.3%) were grouped as having severe dengue based on the 2009 WHO definitions. The time from onset of dengue illness to CRP measured ranged from 1 to 5 days (median, 3 days). Among these 6 patients, acute kidney injury developed in 4 (66.7%), severe hepatitis and gastrointestinal bleeding each developed in one (16.7%), and 2 (30%) died of DSS.

### 3.3. CRP Levels Stratified by Febrile, Critical, and Convalescent Phases

Of the 191 patients with dengue, 97 had CRP levels measured during their febrile phase, 85 during critical phase, and 9 during convalescent phase. By 1997 WHO dengue severity classification, the median CRP level was significantly higher for DSS versus DF (124.5 mg/L versus 13.8 mg/L; *P* < 0.001); DSS versus nonshock DHF (124.5 mg/L versus 15.8 mg/L; *P* = 0.008); DSS versus DF/nonshock DHF (124.5 mg/L versus 21 mg/L; *P* = 0.001) in the febrile phase; and nonshock DHF versus DF (9.9 mg/L versus 6 mg/L; *P* = 0.016) in the critical phase (Table 4). According to the 2009 WHO dengue severity classification, the median CRP level was significantly higher for severe dengue compared to nonsevere dengue during the febrile phase (36.2 mg/L versus 14.4 mg/L; *P* = 0.025) (Table 5).

### 3.4. The Cutoff Level of CRP to Determine DSS and Severe Dengue

The ROC curve analysis was performed for (1) DF versus nonshock DHF/DSS, (2) DF/nonshock DHF versus DSS, and (3) nonsevere dengue versus severe dengue. Regardless of the phases of illness, for DF versus nonshock DHF/DSS and DF/nonshock DHF versus DSS, the AUC was 0.652 and 0.806, respectively. This demonstrates that CRP is insufficient to differentiate between DF and nonshock DHF/DSS. In contrast, CRP was a good discriminant between DF/nonshock DHF and DSS, with CRP cutoff level of 30.1 mg/L, corresponding to 83.3% sensitivity and 81.6% specificity. In the febrile phase of illness, a CRP cutoff level of 30.1 mg/L had 100% sensitivity and 76.3% specificity with AUC of 0.938 for differentiating between DSS and DF/nonshock DHF.

By 2009 WHO dengue severity classification, irrespective of the day of illness, the optimum CRP cutoff level was 24.2 mg/L (0.702 AUC) with 66.7% sensitivity and 76.7% specificity for nonsevere dengue versus severe dengue. In the febrile phase of illness, similarly, a CRP cutoff level of 24.2 mg/L (0.717 AUC) was obtained with 70% sensitivity and 71.3% specificity for differentiating between nonsevere dengue and severe dengue.

## 4. Discussion

CRP is used as an inflammation biomarker, particularly for bacterial infections [23, 24]. Viral infection is commonly assumed to increase CRP concentrations from 10 to 40 mg/L, whereas elevated CRP > 40 mg/L is mainly found in acute bacterial infections [24–26]. In our study, median CRP level was significantly higher in those with nonshock DHF versus DF, DSS versus DF, DSS versus nonshock DHF, DSS versus DF/nonshock DHF, and severe dengue versus nonsevere dengue. Interestingly, the level of CRP was shown to be relatively low in DF and nonsevere dengue patients (median CRP < 10 mg/L), whereas extremely high CRP level was found in patients with DSS (median CRP > 100 mg/L) and severe dengue (median CRP > 30 mg/L). The critical stage of dengue occurs most commonly towards the late febrile phase, usually after the 3rd day of illness [5]. Notably, a significant association of higher CRP levels with DSS and severe dengue was found during the first 3 days of illness (febrile phase) in our series. This finding suggests that CRP is a potentially important simple biomarker in distinguishing patients with severe form of disease from those with mild illness prior to the critical stage of dengue illness.

Our analysis demonstrated that a CRP cutoff level of 30.1 mg/L had an excellent sensitivity (100%) for predicting DSS, whereas a CRP cutoff level of 24.2 mg/L had a sensitivity of 70% for severe dengue, during the first 3 days of illness (febrile phase). The difference in the cutoff level of CRP for DSS and severe dengue can be explained by the different classification schemes for detecting severe cases of dengue [2, 5]. DSS was characterized by signs of circulatory failure associated with the presence of 4 specific criteria (fever, plasma leakage, hemorrhagic manifestations, and thrombocytopenia (≤100 × 10^9^ cells/L)) that met the case definition of DHF [2]. In contrast, the 2009 revised classification used a single criterion (compensated shock, severe bleeding, or severe organ involvement) for defining a severe case, independent of the presence of the 4 criteria to determine disease severity [5]. Actually, the 2009 revised classification is more sensitive in identifying severe disease, and this explains the higher numbers of cases within this group of patients when compared to DSS [27]. Our analysis suggests that using the CRP level with a cutoff level of 24 mg/L during the febrile phase of illness as a criterion for hospital admission is appropriate and that patients with high CRP levels (>30 mg/L) should receive appropriate management as those patients are at higher risk for life-threatening DSS.

In the present study, extremely high CRP (>100 mg/L) levels were noted in 6 patients, with fatal outcomes in 2. Remarkably, organ dysfunction, such as acute kidney injury and severe hepatitis, was found in a majority of patients who had extremely high CRP levels. Higher CRP levels indicate more serious tissue damage resulting from greater inflammation in the body and are often associated with poor outcome [13, 28]. This finding emphasizes that patient with dengue with high CRP levels should therefore be intensively monitored and treated in a timely manner. Bacterial infection was excluded from our study and these findings do not apply to cases of superimposing bacterial sepsis in patients with dengue with high CRP levels.

There are some limitations to our study. First, the small number of DSS and severe dengue cases makes the statistical power quite small. Second, our study excluded patients under 18 years of age. Future prospective studies are needed to validate our findings in different populations for better generalization. Despite the limitations, this study is the first to examine the impact of single CRP testing on clinical decision-making and to evaluate the evidence base for the utility of this test to differentiate mild versus severe illness in adult patients with dengue.

In summary, our study highlights single measurement of CRP as a potential useful and simple biomarker to identify patients who are at risk for developing a more severe dengue illness and to help triage patients requiring hospital care. This information is especially important to clinicians in countries where medical resources are sparse and the burden of dengue is high.

## Figures and Tables

**Table 1:** Patient characteristics.

Variable	*N* = 191
*Demographics and comorbid conditions* ^1^	
Mean age (±SD), years	52.6 (16.0)
Male, *n* (%)	91 (47.6)
Type 2 diabetes mellitus, *n* (%)	41 (21.5)
Essential hypertension, *n* (%)	60 (31.4)
Chronic kidney disease, *n* (%)	11 (5.8)
Previous stroke, *n* (%)	5 (2.1)
*Dengue virus serotype, n/N (%) *	
Serotype 1	5/90 (5.6)
Serotype 2	63/90 (70)
Serotype 3	22/90 (24.4)
Serotype 4	0
*Dengue severity, n (%) *	
1997 WHO classification	
DF	122 (63.9)
Nonshock DHF	63 (32.9)
DSS	6 (3.1)
2009 WHO classification	
Nonsevere dengue	176 (92.1)
Severe dengue	15 (7.9)
*Clinical features at hospital presentation* ^2^	
Mean day from onset illness to presentation (±SD)	3.4 (1.7)
Fever, *n* (%)	182 (95.3)
Abdominal pain, *n* (%)	47 (24.6)
Orbital pain, *n* (%)	26 (13.6)
Bone pain, *n* (%)	69 (36.1)
Myalgia, *n* (%)	104 (54.5)
Headache, *n* (%)	67 (35.1)
Rashes, *n* (%)	38 (20)
Vomiting/nausea, *n* (%)	47 (24.6)
Diarrhea, *n* (%)	43 (22.5)
Petechial, *n* (%)	40 (20.9)
Cough, *n* (%)	47 (24.6)
Dizziness, *n* (%)	43 (22.5)
Gastrointestinal bleeding, *n* (%)	15 (7.9)
Gum bleeding, *n* (%)	23 (12)
*Laboratory features and outcome *	
Leukopenia (WBC < 3.0 × 10^9^ cells/L), *n* (%)	57 (29.8)
Mean hematocrit (±SD) (%), (*n*)	41 ± 4.5 (*n* = 164)
Mean platelet count (±SD) (×10^9^ cells/L), (*n*)	62.6 ± 45.1 (*n* = 189)
Mean CRP (±SD) mg/L	19.3 (27.6)
Fatality, *n* (%)	3 (1.6)

CRP = C-reactive protein; DF = dengue fever; DHF = dengue hemorrhagic fever; DSS = dengue shock syndrome; WBC = white blood cell count; WHO = World Health Organization.

^1^An individual patient might have more than one underlying disease/condition.

^2^An individual patient might have more than one symptom and/or sign.

**Table 2:** CRP level distributions by the 1997 World Health Organization dengue classification.

	DF (*n* = 122)	Nonshock DHF (*n* = 63)	DSS (*n* = 6)	*P* ^1^	*P* ^2^	*P* ^3^	*P* ^4^
Median CRP (range), mg/L	8.5 (0.7–215.5)	15.2 (0.5–139)	124.5 (1.2–205.5)	0.024	<0.001	<0.001	0.011

CRP = C-reactive protein; DF = dengue fever; DHF = dengue hemorrhagic fever; DSS = dengue shock syndrome.

^1^DF versus nonshock DHF.

^2^DF versus DSS.

^3^Nonshock DHF versus DSS.

^4^DF/nonshock DHF versus DSS.

**Table 3:** CRP level distributions by the 2009 World Health Organization dengue classification.

	Nonsevere dengue (*n* = 176)	Severe dengue (*n* = 15)	*P*
Median CRP (range), mg/L	9.8 (0.5–215.5)	30.7 (1.2–205.5)	0.009

CRP = C-reactive protein.

**Table 4:** CRP level distributions by the 1997 World Health Organization dengue classification and phase of illness.

Phase of illness	Median CRP (range)/no, mg/L			*P* ^1^	*P* ^2^	*P* ^3^	*P* ^4^
	DF	Nonshock DHF	DSS				
Febrile phase (days 1–3)	13.8 (0.7–56.2)/61	15.8 (0.6–139.3)/32	124.5 (30.7–205.5)/4	0.070	<0.001	0.008	0.001
Critical phase (days 4–6)	6 (0.8–215.5)/53	9.9 (0.5–71.1)/31	144 (—)/1	0.016	—	—	—
Convalescent phase (days 7–10)	3.3 (1.6–10.7)/8	—	1.2 (—)/1	—	—	—	—

CRP = C-reactive protein; DF = dengue fever; DHF = dengue hemorrhagic fever; DSS = dengue shock syndrome; no = number of patients.

^1^DF versus nonshock DHF.

^2^DF versus DSS.

^3^Nonshock DHF versus DSS.

^4^DF/nonshock DHF versus DSS.

**Table 5:** CRP level distributions by 2009 World Health Organization dengue classification and phase of illness.

Phase of illness	Median CRP (range)/no, mg/L		*P*
	Nonsevere dengue	Severe dengue	
Febrile phase (days 1–3)	14.4 (0.6–69)/87	36.2 (3.3–205.5)/10	0.025
Critical phase (days 4–6)	8 (0.5–215.5)/81	29.2 (6.9–144)/4	0.053
Convalescent phase (days 7–10)	3.3 (1.6–10.7)/8	1.2 (—)/1	—

CRP = C-reactive protein; no = number of patients.
